# Factors affecting the use of neurally adjusted ventilatory assist in the adult critical care unit: a clinician survey

**DOI:** 10.1136/bmjresp-2020-000783

**Published:** 2020-12-08

**Authors:** Daniel Hadfield, Louise Rose, Fiona Reid, Victoria Cornelius, Nicholas Hart, Clare Finney, Bethany Penhaligon, Clare Harris, Sian Saha, Harriet Noble, John Smith, Philip Anthony Hopkins, Gerrard Francis Rafferty

**Affiliations:** 1 Critical Care Research, King's College Hospital, London, UK; 2 King's College London, Centre for Human and Applied Physiological Sciences, London, UK; 3 King's College London Florence Nightingale School of Nursing and Midwifery, London, London, UK; 4 Faculty of Nursing, Midwifery and Palliative Care, King's College, London, UK; 5 King's College London School of Population Health and Environmental Sciences, London, London, UK; 6 Imperial College London School of Public Health, London, London, UK; 7 Centre for Human and Applied Physiological Sciences, King's College London School of Biomedical Sciences, London, UK; 8 Lane Fox Respiratory Unit, Guy's and St Thomas' Hospitals NHS Trust, London, London, UK

**Keywords:** assisted ventilation

## Abstract

**Background:**

Neurally adjusted ventilatory assist (NAVA) involves an intricate interaction between patient, clinician and technology. To improve our understanding of this complex intervention and to inform future trials, this survey aimed to examine clinician attitudes, beliefs and barriers to NAVA use in critically ill adults within an institution with significant NAVA experience.

**Methods:**

A survey of nurses, doctors and physiotherapists in four Intensive Care Units (ICUs) of one UK university-affiliated hospital (75 NAVA equipped beds). The survey consisted of 39 mixed open and structured questions. The hospital had 8 years of NAVA experience prior to the survey.

**Results:**

Of 466 distributed questionnaires, 301 (64.6%) were returned from 236 nurses (78.4%), 53 doctors (17.6%) and 12 physiotherapists (4.0%). Overall, 207/294 (70.4%) reported clinical experience. Most agreed that NAVA was safe (136/177, 76.8%) and clinically effective (99/176, 56.3%) and most perceived ‘improved synchrony’, ‘improved comfort’ and ‘monitoring the diaphragm’ to be key advantages of NAVA. ‘Technical issues’ (129/189, 68.3%) and ‘NAVA signal problems’ (94/180, 52.2%) were the most cited clinical disadvantage and cause of mode cross-over to Pressure Support Ventilation (PSV), respectively. Most perceived NAVA to be more difficult to use than PSV (105/174, 60.3%), although results were mixed when compared across different tasks. More participants preferred PSV to NAVA for initiating ventilator weaning (93/171 (54.4%) vs 29/171 (17.0%)). A key barrier to use and a consistent theme throughout was ‘low confidence’ in relation to NAVA use.

**Conclusions:**

In addition to broad clinician support for NAVA, this survey describes technical concerns, low confidence and a perception of difficulty above that associated with PSV. In this context, high-quality training and usage algorithms are critically important to the design and of future trials, to clinician acceptance and to the clinical implementation and future success of NAVA.

**Key messages**

- What are the factors affecting the use of neurally adjusted ventilatory assist (NAVA) in the adult critical care unit?

- Despite broad clinician support for NAVA, technical concerns, low staff confidence and a perception of technical difficulty are relevant factors that affect the clinical use and adoption of this technology.

- This is the first study to describe the lived experience of clinicians using NAVA, describing factors that are relevant both to future research trials and general clinical implementation.

## Background

Neurally adjusted ventilatory assist (NAVA) uses the electrical activity of the diaphragm (Edi), obtained via a specialised nasogastric feeding catheter (Getinge, Solna, Sweden), as a measure of neural respiratory drive to control the delivery of inspiratory support by a mechanical ventilator (MV).^1^ In 12 years of clinical use, numerous clinical studies have suggested important physiological benefits^2 3^ with recent trials suggesting reduced weaning time^4^ and increased ventilator-free days.^5 6^ Despite this, no trial has yet definitively demonstrated improved patient outcomes and NAVA has not been widely adopted into clinical care.

The implementation of any new technology-based healthcare treatment is complex. Evidence from clinical trials is important, but the success of trials and the subsequent implementation into practice is dependent on a number of other factors. In the case of NAVA, such factors potentially include contextual issues (eg, prevalent culture, cost and access to the technology), human issues (eg, user skill level, training requirements, beliefs and attitudes) and technological issues (eg, performance and limitations of the technology). Feasibility studies are recommended to investigate such factors prior to the conduct of large, resource intensive randomised controlled rials (RCTs), to optimise efficiency and chances of success.^7^

In a recently published trial, we demonstrated the feasibility of evaluating NAVA in an RCT compared with PSV for patients at risk of prolonged ventilation support, with adequate compliance to the assigned ventilatory mode over prolonged durations.^5^ Despite satisfactory mode compliance, some non-adoption and mode cross-over from NAVA to PSV were still observed. To improve our understanding of these cross-over events and wider factors affecting the use of NAVA, we developed and conducted a survey aimed at exploring clinician attitudes, beliefs, perceived barriers and other factors that potentially affect the implementation of NAVA in critically ill adult patients. These data are critical to the design and interpretation of subsequent trials and may help to explain the slow progress towards efficacy and effectiveness trials and clinical adoption of NAVA worldwide.

## Methods

An anonymous, self-administered cross-sectional survey of nurses, doctors and physiotherapists was undertaken in four ICUs (surgical, general medical, neuro/trauma and liver) totalling 75 beds at a university affiliated hospital in London, UK. The survey was conducted alongside a randomised controlled feasibility trial (NCT01826890) comparing NAVA to PSV in adults at risk of prolonged ventilation. The purpose of the study, assurance of anonymity and voluntary nature of the survey was outlined in the participant information sheet. Informed consent was assumed on completion of the questionnaire.

### Survey development and testing

Questionnaire items generated following an evidence review and previous qualitative work,^8^ were refined in three phases through (1) expert review, (2) cognitive interviews and (3) pilot survey distribution. Items were then revised and reduced by an expert team composed of an ICU nurse, a consultant intensivist and an academic physiologist. Questions were formatted into four domains: (1) baseline demographics, NAVA experience and training, (2) beliefs, attitudes and barriers, (3) perceived advantages and disadvantages, and (4) views on NAVA research. Paper-based and web-based (SurveyMonkey) surveys were designed and trialled by a senior statistician and two ICU clinical research nurses in addition to the core expert panel, assessing the clarity, acceptability, time to completion and face validity of the instrument.

Following the process described above, individual cognitive interviews were conducted with three consultant level intensivists, one senior physiotherapist, two senior ICU nurses and two junior ICU nurses. The cognitive interviewing technique is a qualitative method designed to investigate whether a survey question achieves its intended purpose.^9^ Interview notes were collated and used to further revise the draft survey. Finally, electronic and paper surveys were sent to five clinical ICU research nurses, one consultant physiotherapist, and one consultant intensivist to further confirm the time to complete, face and content validity.

### Context

NAVA became a treatment option at the study site in 2008 and the RCT ran between 2012 and 2018. During the trial period, NAVA was used in an estimated 4%–7% of all ventilated patients admitted to the ICU, approximately 60–100 patients per year, 10 of which were recruited to the trial. PSV remained the predominant choice for ventilatory weaning, reflecting ventilatory weaning practice worldwide.^10^ NAVA was applied mostly in patients with risk factors for prolonged weaning (study inclusion criteria), but also in a range of clinical circumstances outside of the trial. Treatment plans were made during daily, physician-led, multidisciplinary ward rounds; changes to ventilation (eg, mode or support-level changes) were made by both nurses and doctors. Physiotherapists were involved in assessment, planning and adjustment of ventilation in complex and difficult to wean patients. NAVA education was offered to all staff during scheduled, general training events and individual sessions were provided as needed. Although not specified in the survey instrument, due to the almost exclusive use of NAVA in intubated patients, it was implicit that the survey was addressing issues around invasive ventilation.

### Sampling frame

The sample comprised staff with responsibility for the management of MV at the study site. A database containing contact details, professions and grades was accessed with appropriate local permissions. The target population consisted of 365 nurses, 89 doctors and 12 physiotherapists.

### Instrument administration

The electronic questionnaire (online supplemental file 1) was administered to all eligible staff on the 15 May 2017, towards the end of the clinical trial that ended in January 2018. Non-responders received up to three additional reminder emails, with the final reminder sent on 12 July 2017. The final response was received on 24 July 2017 when the survey was closed. Paper questionnaires were delivered to all ICU staff who had not completed an electronic survey; participants were checked off a master list to avoid duplication. All questionnaires were self-administered. No incentive was offered to encourage participation.

10.1136/bmjresp-2020-000783.supp1 Supplementary data

### Statistics and analysis

As the project was primarily descriptive in nature a power calculation was not appropriate as hypothesis testing was not a main aim. Statistical analyses were performed using GraphPad Prism (V.8.0 for Windows, GraphPad Software, San Diego, California, USA).^11^ The response rate was calculated as the total number of returned surveys with answered questions, including those that were partially completed, divided by the total number of eligible participants identified at study start. Descriptive statistics are presented, including count, percentage and 95% confidence intervals (CIs), or medians with inter quartile ranges (IQRs) as appropriate. Five or seven-point Likert scales^12^ and multiple options questions were used to assess attitudes, opinions and agreement with statements. Ordinal Likert data were converted to ranks; descriptive data are presented as median ranks, and Spearman’s rank-order correlation was used to assess association. Distributions of categorical variables were compared using the χ^2^ test or Fisher exact test. Free-text ‘other’ options were included in all multiple-options questions from which recurring themes were identified.

### Patient and public involvement

Patients or the public were not involved in the design of the study.

## Results

Of the 466 questionnaires distributed, 301 (64.6%) were returned including responses from 236 nurses (78.4%), 53 doctors (17.6%) and 12 physiotherapists (4.0%). The response rate expressed as a proportion of the eligible staff was 64.7% for nurses, 59.6% for doctors and 100.0% for physiotherapists. Responses were obtained across all levels of seniority; most participants were junior grade (72.7%), aged 25–34 years (56.0%), and with less than 3 years of ICU experience (60.8%) (table 1), which reflected the general profile of ICU staff.

**Table 1:** Survey distribution, response, demographics and experience of NAVA

Characteristic	All staff	Nurses	Doctors	Physiotherapists
**Years in KCH ICU**				
<1	***96/301 (31.9)***	70/236 (30.0)	18/53 (35.9)	7/12 (58.3)
1–3	***87/301 (28.9)***	71/236 (30.1)	15/53 (28.3)	1/12 (8.3)
3–5	***49/301 (16.3)***	41/236 (17.4)	5/53 (9.4)	3/12 (25.0)
>5	***69/301 (22.9)***	54/236 (22.9)	14/53 (26.4)	1/12 (8.3)
Seniority*				
Junior	***193/297 (65.0)***	171/235 (72.8)	19/50 (38.0)	3/12 (25.0)
Middle grade	***54/297 (18.1)***	39/235 (16.6)	11/50 (22.0)	4/12 (33.3)
Senior	***50/297 (16.8)***	25/235 (10.6)	20/50 (40.0)	5/12 (41.7)
Familiarity with key concepts*^†^				
Risk factors for PMV	***289/294 (98.3)***	225/229 (98.3)	52/53 (98.1)	12/12 (100.0)
Evidence for PSV	***269/292 (92.1)***	211/227 (93.0)	46/53 (86.8)	12/12 (100.0)
Evidence for NAVA	***163/291 (56.0)***	120/226 (53.1)	36/53 (67.9)	7/12 (58.3)
Training and experience*				
NAVA trained	***157/295 (53.2)***	124/230 (53.9)	28/53 (52.8)	5/12 (41.6)
Any NAVA use	***207/294 (70.4)***	153/229 (66.8)	47/53 (88.7)	7/12 (58.3)
Approximate no of patients treated where NAVA was used*				
<5	***143/287 (49.8)***	115/223 (51.6)	24/53 (45.3)	4/11 (36.4)
5–20	***62/287 (21.6)***	45/223 (20.2)	15/53 (28.3)	2/11 (18.2)
>20	***16/287 (5.6)***	5/223 (2.2)	10/53 (18.9)	1/11 (9.1)
None	***66/287 (23.0)***	58/223 (26.0)	4/53 (7.6)	4/11 (36.4)
Frequency of NAVA use *‡				
Use in past week?	***5/193 (2.6)***	2/141 (1.4)	3/45 (6.7)	0/12 (0.0)
Use in past month?	***16/193 (8.3)***	6/141 (4.3)	10/45 (22.2)	0/12 (0.0)
Use >1 month prior	***164/193 (85.0)***	125/141 (64.8)	32/45 (71.1)	7/12 (58.3)

Data are number (%). Nurse and physiotherapist grade according to UK Agenda for Change (AFC) grades: Junior=AFC band 5; Middle=AFC band 6; Senior=AFC bands seven and above.

Doctors grades according to UK medical role classifications. Junior=foundation year 1/2 or equivalent; Middle=junior or senior middle grades (eg, specialty trainees, clinical fellows, senior house officers); senior=consultant level.

Bold/italic values represent the overall response rate, including all professions

*Numbers do not total 301 because not all participants answered every question. Denominators provided.

†Assessed on a five-point Likert scale: Not at all, slightly, moderately, very and extremely familiar. The presented data are the combined proportions answering either slightly, moderately, very or extremely familiar. See online supplemental file 2 for response numbers in each category.

‡Only staff reporting clinical exposure to NAVA were asked to complete this and subsequent questions.

KCH ICU, King's College Hospital Intensive Care Unit; NAVA, neurally adjusted ventilatory assist; PMV, prolonged mechanical ventilation; PSV, pressure support ventilation.

10.1136/bmjresp-2020-000783.supp2 Supplementary data

Of all participants, 157/295 (53.2%) had received NAVA training, 207/294 (70.4%) reported clinical exposure to NAVA (at least one patient treated where NAVA was used), 163/291 (56.0%) indicated that they were familiar (slightly, moderately, very or extremely) with evidence supporting NAVA use in weaning, 269/292 (92.1%) with evidence supporting PSV in weaning, and 289/294 (98.3%) with risk factors for prolonged MV (table 1 and online supplemental figure S1). Of those reporting clinical exposure to NAVA, 125/193 (64.8%) indicated use of Edi monitoring (online supplemental figure S2) and 110/180 (61.1%) had participated in the concurrent clinical trial. Of those who had received NAVA training, the majority (140/157, 89.2%) received individual bedside training from local staff. Doctors were more likely than nurses or physiotherapists to have used NAVA clinically, in greater than five patients and recently (within the month prior to the survey), perhaps reflecting their role in medical management of multiple patients compared with nursing management of individual patients (table 1). Doctors were also more likely to indicate familiarity with NAVA evidence compared with nurses or physiotherapists (p=0.006). Overall, very few staff had used NAVA in the week (5/185, 2.6%) or month (16/185, 8.3%) prior to the survey, but most had used NAVA within the past 6 months (113/185, 61.1%) (table 1).

Only those reporting clinical exposure to NAVA (as opposed to training experience) were asked to proceed to subsequent questions.

Most participants agreed or strongly agreed that NAVA was safe (136/177, 76.8%), and clinically effective (99/176, 56.3%), that diaphragm monitoring was clinically effective (101/172, 58.7%), and that ventilator dysynchrony was a clinically significant issue (133/174, 60.3%) (table 2 and online supplemental figure S3). When asked about general feelings, 94/179 (52.5%) indicated ambivalence, 59/179 (33.0%) ‘liked’ (slightly, moderately or strongly) and 26/179 (14.5%) ‘disliked’ (slightly, moderately or strongly) NAVA, suggesting good equipoise for future randomised trials (online supplemental figure S4). In comparison to PSV, similar numbers of participants perceived better (slightly, moderately or significantly) (43/190, 22.6%), worse (slightly, moderately or significantly) (37/190, 19.5%), or equivalent (49/190, 25.8%) NAVA clinical performance, with 61/190 (32.1%) indicating ‘don’t know’, and with no significant differences between professional groups (online supplemental figure S5). More participants indicated a preference for PSV (93/171, 54.4%) compared with NAVA (29/171, 17.0%) for initiation of ventilatory weaning, although a large proportion (49/171, 27.1%) were ambivalent.

**Table 2:** General beliefs and attitudes towards NAVA (participants with clinical experience only)

Question and response	All staff	Nurses	Doctors	Physio
**General feelings towards NAVA**				
Slightly/moderately/strongly like	***59/179 (33.0)***	43/131 (32.8)	13/41 (31.7)	3/7 (42.9)
Ambivalent	***94/179 (52.5)***	68/131 (51.9)	24/41 (58.5)	2/7 (28.6)
Slightly/moderately/strongly dislike	***26/179 (14.5)***	20/131 (15.3)	4/41 (9.8)	2/7 (28.6)
**Perceived performance compared with PSV**				
Slightly/moderately/significantly better	***43/190 (22.6)***	26/138 (18.8)	16/45 (35.6)	1/7 (14.3)
Equivalent	***49/190 (25.8)***	38/138 (27.5)	8/45 (17.8)	3/7 (42.9)
Slightly/moderately/significantly worse	***37/190 (19.5)***	27/138 (19.6)	9/45 (20.0)	1/7 (14.3)
Don’t know	***61/190 (32.1)***	47/138 (34.1)	12/45 (26.7)	2/7 (28.6)
**NAVA is safe (agreement)**				
Agree/strongly agree	***136/177 (76.8)***	97/129 (75.2)	32/41 (78.1)	7/7 (100.0)
Ambivalent	***32/177 (18.1)***	31/129 (24.0)	8/41 (19.5)	0/7 (0.0)
Disagree/strongly disagree	***7/177 (4.0)***	1/129 (1.0)	1/41 (2.4)	0/7 (0.0)
**NAVA is clinically effective (agreement)**				
Agree/strongly agree	***99/176 (56.3)***	70/129 (54.3)	28/40 (70.0)	1/7 (14.3)
Ambivalent	***70/176 (39.8)***	53/129 (41.1)	11/40 (27.5)	6/7 (85.7)
Disagree/strongly disagree	***7/176 (4.0)***	6/129 (4.7)	1/40 (2.5)	0/7 (0.0)
**Edi monitoring is clinically effective (agreement)**				
Agree/strongly agree	***101/172 (58.7)***	72/125 (57.4)	25/40 (62.5)	4/7 (57.1)
Ambivalent	***60/172 (34.9)***	44/125 (34.1)	13/40 (32.5)	3/7 (42.9)
Disagree/strongly disagree	***11/172 (6.4)***	9/125 (7.0)	2/40 (5.0)	0/7 (0.0)
**Dysynchrony is clinically significant (agreement)**				
Agree/strongly agree	***133/174 (76.4)***	96/127 (75.6)	31/40 (77.5)	6/7 (85.7)
Ambivalent	***32/174 (18.4)***	24/127 (18.9)	7/40 (17.5)	1/7 (14.3)
Disagree/strongly disagree	***7/174 (4.0)***	5/127 (3.9)	2/40 (5.0)	0/7 (0.0)
**NAVA is more difficult than PSV (agreement)**				
Agree/strongly agree	***105/174 (60.3)***	69/127 (54.3)	31/40 (77.5)	5/7 (71.4)
Ambivalent	***40/174 (23.0)***	36/127 (28.3)	3/40 (7.5)	1/7 (14.3)
Disagree/strongly disagree	***29/174 (16.7)***	22/127 (17.3)	6/40 (15.0)	1/7 (14.3)
**Preferred mode in ventilatory weaning**				
NAVA	***29/171 (17.0)***	21/124 (16.9)	8/40 (20.0)	0/7 (0.0)
PSV	***93/171 (54.4)***	64/124 (51.6)	23/40 (57.5)	6/7 (85.7)
No preference	***49/171 (28.8)***	39/124 (31.5)	9/40 (22.5)	1/7 (14.3)
**Increased workload due to NAVA?**				
None	***85/188 (45.2)***	66/137 (48.2)	14/44 (31.8)	5/7 (71.4)
Slight/moderate increase	***69/188 (36.7)***	51/137 (37.2)	16/44 (36.4)	2/7 (28.6)
Large/substantial increase	***5/188 (2.7)***	1/137 (1.0)	4/44 (9.1)	0/7 (0.0)
Don’t know	***29/188 (15.4)***	19/137 (13.9)	10/44 (22.7)	0/7 (0.0)

Data are number (%). See online supplemental file 2 for response numbers in each category.

Bold/italic values represent the overall response rate, including all professions

Edi, electrical activity of the diaphragm; NAVA, neurally adjusted ventilatory assist.

**Figure 1:**
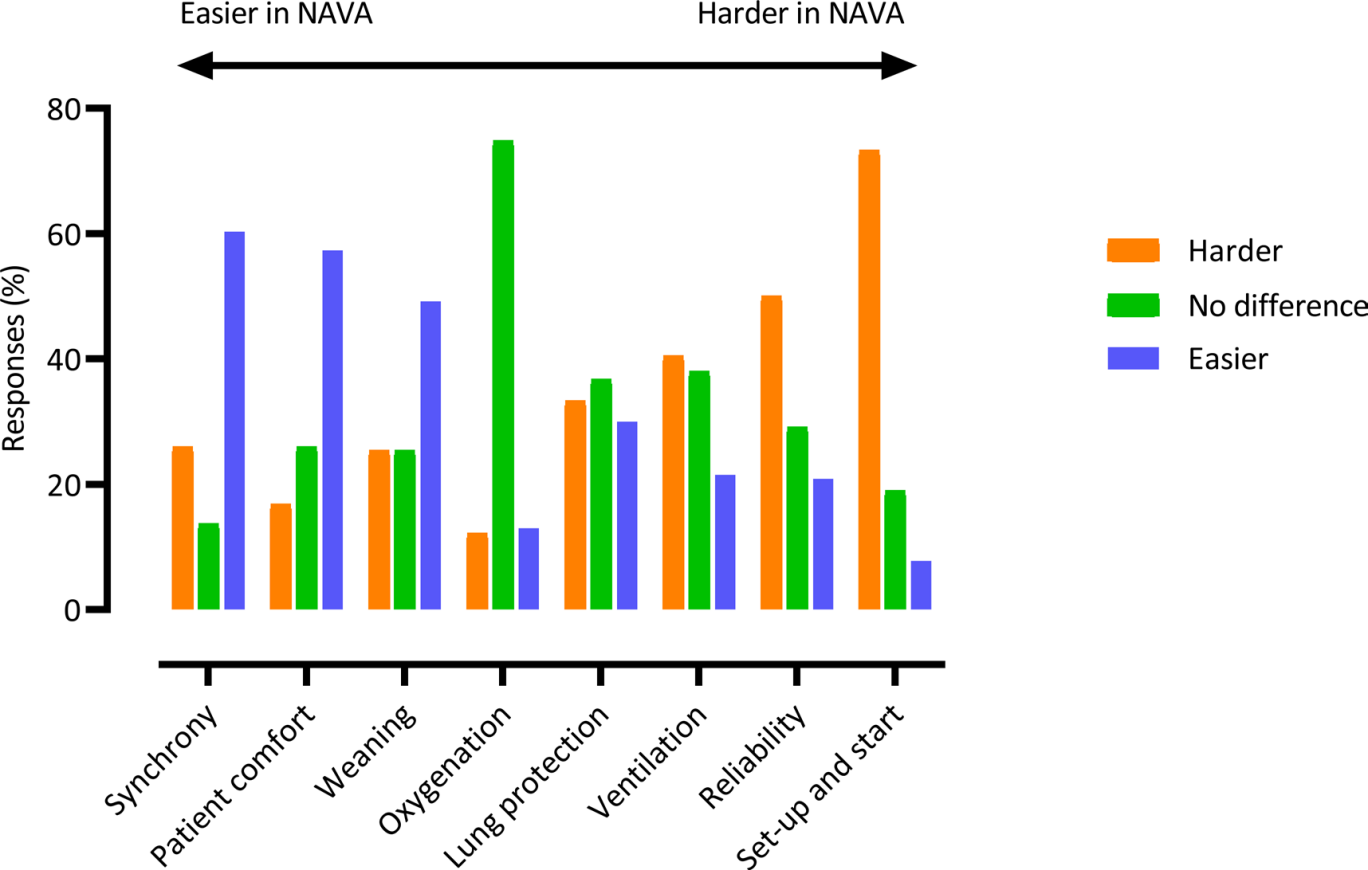
How easy is it to achieve the following aspects of ventilation practice when using the NAVA mode compared to the PSV mode? Assessed on a seven-point Likert scale: much, moderately, slightly easier; no difference; slightly, moderately, much harder. see online supplemental file 2 for individual response rates for each category and a breakdown of responses in each category. For ease of interpretation, activities are ordered from left to right broadly in terms of the difficulty of each activity in NAVA compared with PSV as indicated by the horizontal arrow, with oxygenation and lung protection central due to the relative equipoise of participants. NAVA, neurally adjusted ventilatory assist; PSV, pressure support ventilation.

Overall most participants indicated that NAVA was more difficult than PSV (105/174, 60.3%) (table 2), and considered it was harder (much/moderately/slightly) rather than easier (much/moderately/slightly) in relation to ‘set up and start’ (85/116 (73.3%) vs 9/116 (7.8%)), ‘ventilation’ (49/121 (40.5%) vs 26/121 (21.5%)) and ‘reliability’ (55/110 (50.0%) vs 23/110 (20.9%)) (figure 1 and online supplemental figure S6). More participants indicated that NAVA was easier than PSV, rather than harder, in relation to ‘synchrony’ (79/131 (60.3%) vs 34/131 (26.0%)), ‘patient comfort’ (75/131 (57.3%) vs 22/131 (16.8%)) and ‘weaning’ (62/126 (49.2%) vs 32/126 (25.4%)). The most frequent response was ‘no difference’ between the two modes in relation to ‘lung protection’ (44/120, 36.7%) and ‘oxygenation’ (92/123, 74.8%). NAVA was not perceived to increase workload by 85/188 (45.2%) of participants, while 74/188 (39.4%) perceived either a slight, moderate, large or substantial workload increase, and with no differences between professional groups (table 2 and online supplemental figure S7).

**Figure 2:**
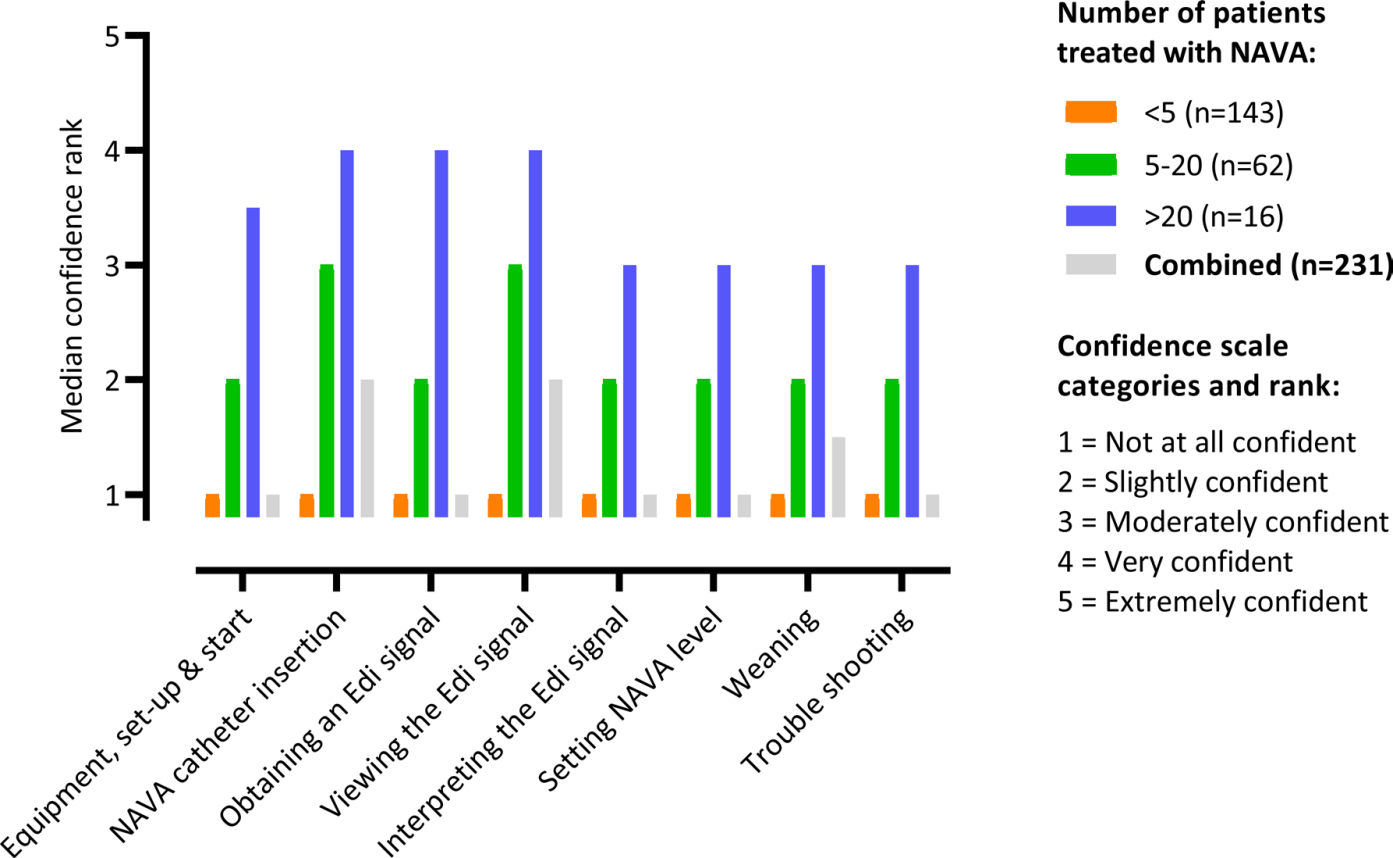
Confidence in performing NAVA tasks compared between staff with differing NAVA experience. Colours refer to different numbers of patients treated where NAVA was used. See online supplemental file 2 for individual response rates for each category. NAVA, neurally adjusted ventilatory assist.

Participants reported low confidence in performing eight NAVA related tasks (median ranks range from 1 to 2 across all tasks); those with greater NAVA exposure in terms of patients treated, were more likely to indicate greater confidence (r=0.562, 95% CI 0.453 to 0.654) (figure 2 and online supplemental figure S8). Level of NAVA exposure was related to years of ICU experience; participants with more years ICU experience were more likely to have used NAVA in a greater number of patients (r=0.531, 95% CI 0.442 to 0.610). Doctors expressed greater confidence compared with nurses, who were consistently more likely to reply, ‘not at all confident’.

**Figure 3:**
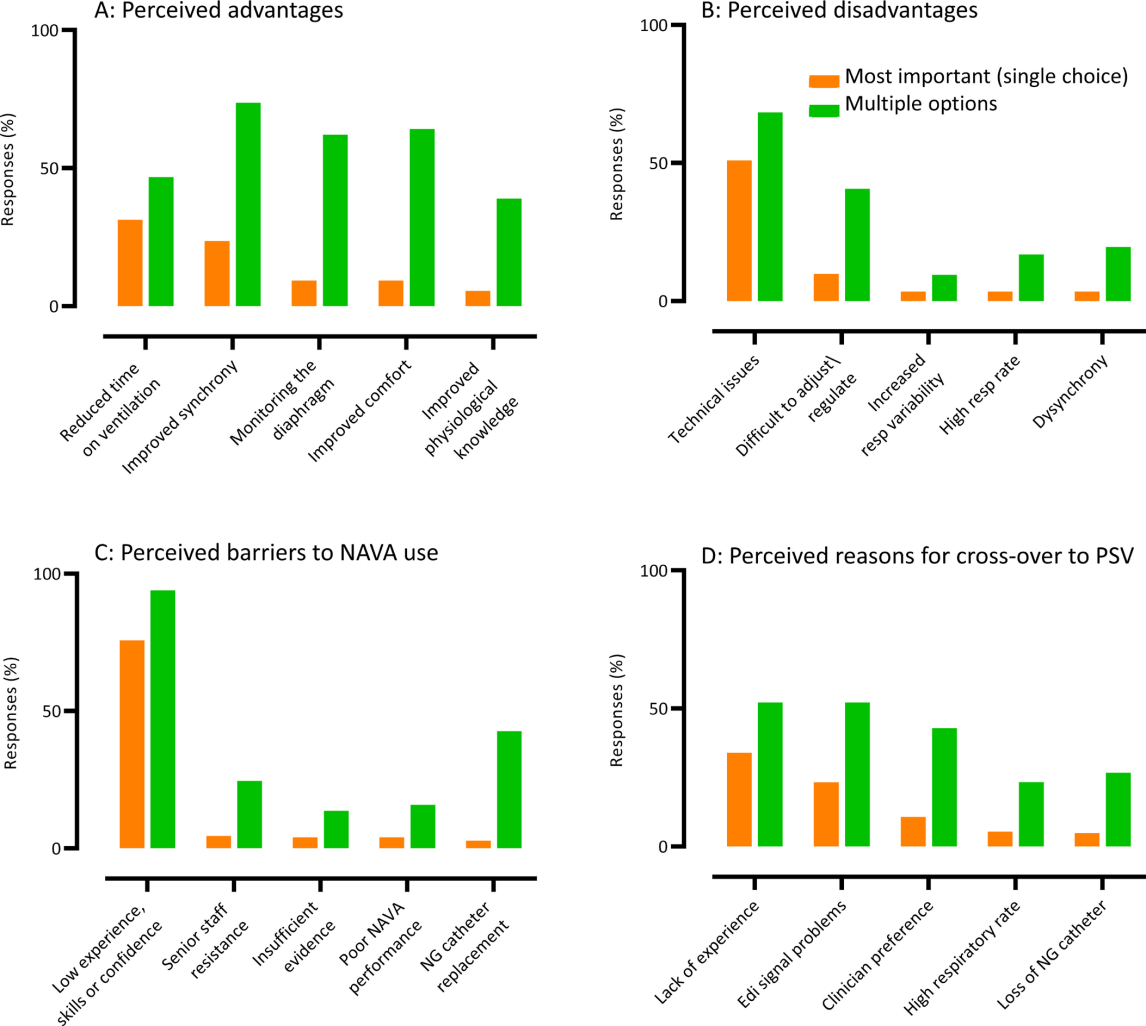
(A). Perceived advantages of neurally adjusted ventilatory assist (NAVA) compared with pressure support ventilation (PSV): multiple options (response rate 190) and single most important (response rate 182). (B) Perceived disadvantages of NAVA compared with PSV: multiple options (response rate 189) and single most important (response rate 173). (C) Barriers to the acceptance and implementation of NAVA: multiple options (response rate 183) and single most important (response rate 177). (D) Reasons for mode cross-over: multiple options (response rate 180) and single most important (response rate 168). Top five single most important reasons presented only. Please see online supplemental file 2 for full a full list of responses.

When asked about the potential benefits of using NAVA and participants could select multiple options, the most common response (140/190, 73.7%) was ‘improved synchrony’ (figure 3 and online supplemental figure S9. The single most important benefit (single choice) was ‘reduced time in ventilation’ (57/183, 31.3%). The need to improve ventilator synchrony and weaning were also considered the most common clinical reasons for NAVA use (133/192 (69.3%) and 99/192 (51.6%), respectively). In regard to the disadvantages of NAVA (figure 3 and online supplemental figure S10), most selected ‘technical issues’ (129/189, 68.3%), with half (88/173, 50.9%) selecting this option as the most important single disadvantage. The next most commonly selected disadvantage was ‘difficult to adjust or regulate’, further suggesting the perception of difficulty and/or complexity with this mode. There were no significant differences between nurses and doctors in their perception of clinical advantages or disadvantages.

The most commonly selected barrier to acceptance and implementation of NAVA, was ‘low experience, skills or confidence’ (172/183, 94.0%) (figure 3 and online supplemental figure S11), which was also selected as the single main barrier (132/171, 78.4%). Most participants believed that in their experience of NAVA, the mode was ‘often switched’ to PSV (56/182, 30.8%), with only very few (19/182, 10.4%) believing the mode was ‘switched rarely’ or ‘not at all’ (online supplemental figure S12). The reasons most often selected for switching were ‘lack of experience’ and ‘Edi signal problems’, which were also the most selected single main reasons for cross-over (57/168, 33.9% and 39/168, 23.2%, respectively) (figure 3 and online supplemental figure S13). When asked what single initiative would most help acceptance and use of NAVA, the majority (101/167, 60.5%) selected ‘improved training and sharing of the evidence base’ (online supplemental figure S14).

When asked about research aimed at investigating the use of NAVA in prolonged MV weaning, 161/180 (89.4%) of participants were supportive, 3/180 (1.7%) were not supportive, and 16/180 (8.9%) did not know. ‘Research evidence’ was considered to be less influential on clinicians’ views on NAVA (21/174, 12.1%) compared with ‘personal, clinical experience’ (93/174, 53.5%) and ‘colleague/peer recommendation’ (54/174, 31.0%).

Out of 42 participants who made a qualitative comment, 10 (23.8%) were supportive of NAVA and/or NAVA research, 23 (54.8%) stated that more training was needed, and 18 (42.9%) highlighted a lack of clinical experience. The following statements are representative and support other data presented above:

1. Nurse: ‘I found that when I had more exposure to NAVA, I became more confident and could see the benefits. But I found the inconsistency made it difficult to use. Sometimes I found it worked really well and other times very difficult to use.’
2. Doctor: ‘In theory NAVA sounds great. I wish I had more experience with it.’
3. Nurse: ‘NAVA is a great idea, but more training needed for staff confidence and being able to troubleshoot.’

## Discussion

This multidisciplinary survey of 301 critical care clinicians, conducted across four adult ICUs at one academic hospital with >8 years of NAVA experience, sought to describe attitudes, beliefs, barriers and other factors relating to NAVA use, both in usual clinical practice and within clinical trials. In summary, the results indicate: (1) supportive attitudes to NAVA and belief that NAVA is safe and clinically effective with broadly equivalent performance to PSV, indicating good equipoise for future research; (2) a perception of increased difficulty and/or complexity compared with PSV; (3) a perception of low experience, skills and confidence, despite reasonable numbers of participants having clinical exposure to NAVA, and a need for improved training and (4) broad support for future NAVA research. These results provide context and feasibility data that can benefit clinical implementation of NAVA and the design of future trials. Perceived increased difficulty of using NAVA, low confidence of clinical staff and technical issues, may help to understand the current disparity between NAVA studies suggesting clinical benefit to patients, and the slow progress towards adequately powered efficacy or effectiveness trials and potential clinical adoption worldwide.

Technical difficulties with the equipment and/or Edi signal acquisition was the most often selected clinical disadvantage and reason for mode cross-over from NAVA to PSV. Cross-over is potentially a significant issue in future trials; low compliance will impact on statistical power and interpretation of results in a definitive study.^13^ Similar issues were not reported in three recent clinical trials,^3 6 14^ however, Edi signal problems were a stated cause of cross-over in 10 out of 36 participants (27.8%) in the concurrent feasibility RCT,^5^ and Edi synchrony and/or signal issues were reported in seven out of 20 (35%) patients recruited to an RCT by Di Mussi *et al*.^15^ Reasons for the differences between these trials remain unclear. Issues may relate to one or a combination of component failures, usability issues, or user behaviour, and these factors may be further influenced by environmental factors, such as staff to patient ratios, admission rates, availability of hardware, and varying levels of clinical expertise across a large staff group. In the NAVA mode, the Edi triggers and controls the support during the respiratory cycle, therefore acquisition of a stable, reliable signal is essential. Although trials have tested the catheter positioning technique,^16 17^ and demonstrated the reliability of the signal in different clinical situations,^1 18–22^ these studies were small (n<20), using cross-over designs with observations of limited durations—usually around 30 min. The results of this survey suggest a need to explore catheter positioning, signal acquisition and signal stability over prolonged periods and in a variety of clinical situations where the neural respiratory drive may become unreliable.

Despite >70% of participants indicating clinical exposure to NAVA and despite >8 years of NAVA use at the study site, perceived low confidence and/or inexperience of clinicians when using NAVA is a central theme within the survey. This finding was also detected in the concurrent feasibility RCT, where clinical inexperience of the mode was considered the reason for cross-over to PSV for four (11%) of 36 participants randomised to NAVA.^5^ Infrequent application within the difficult-to-wean patient group may be relevant to these perceptions, but the results also suggest perceived difficulty and/or complexity of NAVA and technical difficulties in relation to the Edi signal, both likely to impact use and confidence with NAVA.

In the context of a clinical trial, these issues may reduce the fidelity of the NAVA intervention and threaten the internal validity of the results. They may also significantly impact clinical implementation; clinical staff are unlikely to choose a more complex, difficult and novel treatment where an easier existing option that is not perceived to cause harm is available. A comparison may be made to the exponential increase in use of nasal high flow internationally despite a lack of evidence of efficacy,^23^ which may be partially due to its ease of use.

Critically, these factors are potentially modifiable. Our results suggest a need for high quality algorithms, training and standard operating procedures to mitigate these risks. Such approaches would improve the potential of demonstrating a true effect in a clinical trial by ensuring the fidelity of the intervention.^13 24^ Given the limited influence of current NAVA evidence on clinician behaviour suggested here, combined with growing evidence of clinical benefit from NAVA in preliminary studies,^4 5^ a well-designed clinical trial to demonstrate efficacy and/or effectiveness is required and is necessary to prompt behavioural change and clinical implementation.

### Strengths and weaknesses

To the authors’ knowledge, this is the first detailed clinician survey to assess the use of NAVA in the context of a randomised feasibility trial, with a representative range of doctor, nurse, and physiotherapist knowledge and a high response rate from all groups. The main limitation of this work relates to its conduct at a single site meaning results may not be generalisable. In particular, variability in MV management and practice across professions, institutions and healthcare systems, may affect the generalisability of these results. At the study site and in common with other UK sites,^25^ the management of MV is collaborative, with nurses, doctors and physiotherapists requiring knowledge of ventilation modes. This may not be true in other healthcare systems outside of the UK.

As a self-report survey, various biases may have affected participant responses. Despite 44% of the participants being unfamiliar with evidence to support the use of NAVA in weaning, participant opinions may have been biased towards a more positive view (information bias) regarding the efficacy and potential advantages of NAVA due to a largely positive body of literature. Social desirability bias may have influenced participants towards responses that were supportive of NAVA. Data relating to disadvantages and barriers are less susceptible to these biases and are, therefore, potentially of greater value.

In addition, the survey did not assess the knowledge of the participants in relation to the use of PSV or NAVA. Frequent use and greater familiarity with PSV may have affected staff attitudes towards that mode of ventilation. However, despite its relative ease of application^26^ and ubiquitous use, it does not necessarily follow that PSV is correctly understood and applied, or that NAVA was poorly understood and applied. It may be argued that PSV requires less deliberate thought, whereas NAVA demands an understanding of underlying physiology, which may enhance the performance of the user and increase quality of the intervention.^27^

Finally, although this study used ‘the number of patients treated where NAVA was used’ as a surrogate for clinical experience, the level of involvement of each participant in the management of NAVA was not assessed.

## Conclusions

This study presents the experience and views of a large and diverse staff group, describing attitudes, beliefs, perceived barriers and other factors that potentially affect NAVA use in critically ill adult patients. The central perceptions of low confidence, increased difficulty and technical issues reported here, may partially explain slow progress towards clinical implementation and large trials. In this context, high-quality training, algorithms and evidence from large definitive trials are even more critical to clinician acceptance and the future success of NAVA.
