# Supplementary material for: Factors affecting the use of neurally adjusted ventilatory assist in the adult critical care unit: a clinician survey
Source: BMJ Open Respir Res. 2020 Dec 8;7(1):e000783. doi: 10.1136/bmjresp-2020-000783 (PMC7725091; doi:10.1136/bmjresp-2020-000783)
Supplement: Supplementary data [file bmjresp-2020-000783supp001.pdf]

Supplemental file 1: Survey instrument

Manuscript title

Factors affecting the use of Neurally Adjusted Ventilatory Assist in the adult critical care unit: A clinician survey

Corresponding Author

Daniel Hadfield. Email: daniel.hadfield@nhs.net.

BACKGROUND

1. What is your professional group?

Nurse	Doctor	Physiotherapist
○	○	○

2. Current Agenda for Change grade/band (nurses and physiotherapists)

5	6	7	8+
○	○	○	○

3. Current medical grade / level (if applicable)

FY1/2	Junior resident	Senior Resident (809,927,699)	Consultant
○	○	○	○

4. Current job title ....

5. What is your age?

<25	25-34	35-44	45-54	55-64	>64
○	○	○	○	○	○

6. For how long have you worked in the ICU at KCH?

< 1 year	1 - 3 years	3-5 years	>5 years
○	○	○	○

TRAINING AND KNOWLEDGE

7. What form of NAVA training have you received? ***Tick ALL that apply***

A	None	○
B	Group training from the Maquet representative	○
C	Group training from local staff	○
D	Bedside training from local staff / peer to peer	○
E	Maquet website tutorial	○

F	Other (Please state)	○

8. What form of training did you find most useful? ***Please enter the relevant letter from the list above***

9. How would you rate the strength of your general understanding of mechanical ventilation in critically ill adults?

Tick ONE option

Much below average	Below average	Average	Above average	Much above average
○	○	○	○	○

10. Please indicate your level of familiarity with the following:

	Not at all familiar	Slightly familiar	Moderately familiar	Very familiar	Extremely familiar
A: Evidence supporting the use of sedation holds and breathing trials	○	○	○	○	○
B: Risk factors for prolonged ventilatory weaning	○	○	○	○	○
C: The current RESTUS / NAVA trial	○	○	○	○	○
D: Evidence supporting the use of Pressure Support (PS) during weaning	○	○	○	○	○
E: Evidence supporting NAVA use during weaning	○	○	○	○	○

11. Have you been involved directly or indirectly in the care of a patient where NAVA was used?

Yes	No
○	○

12. Of all the patients that you have cared for at KCH, in how many was NAVA used?

≤ 5 patients	5 to 20 patients	> 20 patients	NONE
○	○	○	○

13. Have you had experience of NAVA in any other hospital?

Yes	No	If yes, approximately how many patients?
○	○	

CLINICAL EXPERIENCE OF NAVA

14. How recently have you cared for a patient where NAVA was used?

Within the last week	Within the last month	Within the last 6 months	Within the last year	>1 year	Don't know
o	o	o	o	o	o

15. How confident are you in performing the following NAVA related tasks?

	Not at all confident	Slightly confident	Moderately confident	Very confident	Extremely confident	NA
A: Equipment selection, set-up and initiation	o	o	o	o	o	o
B: NAVA catheter insertion	o	o	o	o	o	o
C: NAVA catheter positioning to obtain diaphragm signal	o	o	o	o	o	o
D: Viewing the diaphragm signal (Edi)	o	o	o	o	o	o
E: Interpreting the diaphragm signal (Edi)	o	o	o	o	o	o
F: Setting the support level	o	o	o	o	o	o
G: Weaning	o	o	o	o	o	o
H: Trouble shooting	o	o	o	o	o	o

16. How have you used the EDI signal? ***Tick ALL that apply***

Viewed as a trend across a period of time	o
To monitor and/or improve synchrony	o
To evaluate readiness for a spontaneous ventilation mode	o
To evaluate readiness for extubation	o
To measure response to interventions (e.g. spontaneous breathing trials, sedation holds, physiotherapy interventions)	o
I have not used the Edi signal	o
Other (Please state)	o

17. Outside of the current trial, what are the most common reasons for NAVA catheter insertion?

Tick ALL that apply

To accelerate weaning	o

To improve synchrony	○
To diagnose muscle dysfunction	○
To monitor the diaphragm	○
Clinician preference	○
Don't know	○
Other (Please state)	○

18. Outside of the current trial, **WHEN** are NAVA catheters most often inserted at KCH? ***Tick ONE option***

At or soon after intubation	At the start of weaning	In later weaning	Don't know
○	○	○	○

19. How ***easy*** is it to achieve the following when using the **NAVA** mode compared to the **PS** mode?

Please record NA if you have no experience of the specific practice

	Much harder in NAVA	Moderately harder in NAVA	Slightly harder in NAVA	No difference	Slightly easier in NAVA	Moderately easier in NAVA	Much easier in NAVA	NA/ don't know
A: Set-up and start the mode	○	○	○	○	○	○	○	○
B: Ventilation (adequate MV and CO_2 clearance)	○	○	○	○	○	○	○	○
C: Lung protection (TV 6-8mls/kg)	○	○	○	○	○	○	○	○
D: Oxygenation	○	○	○	○	○	○	○	○
E: Synchrony	○	○	○	○	○	○	○	○
F: Patient comfort	○	○	○	○	○	○	○	○
G: Weaning	○	○	○	○	○	○	○	○
H: Maintaining the mode without switching (reliability)	○	○	○	○	○	○	○	○

20. **In your experience**, how did NAVA perform clinically in comparison to Pressure Support?

Significantly worse	Moderately worse	Slightly worse	Equivalent	Slight better	Moderately better	Significantly better	Don't know / NA
○	○	○	○	○	○	○	○

ADVANTAGES AND DISADVANTAGES

21. What do you consider are the potential clinical ***benefits*** of using NAVA in comparison to Pressure Support? ***Tick ALL that apply***

**A**	Reduced time on ventilation	○
**B**	Improved patient ventilator interaction (synchrony)	○
**C**	Improved gas exchange	○
**D**	Improved patient sleep	○
**E**	Improved patient comfort	○
**F**	Reduced sedation	○
**G**	Increased variability in pressure and tidal volume	○
**H**	Reduced need for external PEEP to off-set intrinsic PEEP	○
**I**	Monitoring of diaphragm activity and respiratory drive	○
**J**	Reduced risk of diaphragm dysfunction	○
**K**	Improved understanding of respiratory physiology and the patient's condition	○
**L**	Don't know	○
**M**	No clinical benefits	
**N**	Other (Please state)	○

22. What do you consider to be the **ONE** most important potential clinical benefit?

***Please enter ONE letter from the list above.* Record NA if you answered 'M'**

23. What do you consider are the potential clinical ***disadvantages*** of using NAVA in comparison to Pressure Support? ***Tick ALL that apply***

**A**	Technical issues (signal quality / equipment malfunction / reliability)	○
**B**	Increased variability in pressure and tidal volume	○
**C**	High tidal volumes and/or inspiratory pressures	○
**D**	Inferior gas exchange	○
**E**	Difficult to adjust or regulate	○
**F**	High respiratory rate	○

**G**	Inferior patient ventilator interaction (synchrony)	○
**H**	Don't know	○
**I**	No clinical disadvantages	○
**J**	Other (Please state)	○

24. What do you consider is the **SINGLE** most important clinical disadvantage?

    Please enter ONE letter from the list above. **Record NA if you answered 'I'**

25. In your experience, is there an increase in **your personal** workload associated with the use of NAVA?

None	Slight	Moderate	Large	Substantial	Don't know
○	○	○	○	○	○

ATTITUDES AND BARRIERS

26. What do you consider are the main barriers to the acceptance and implementation of NAVA?

Tick ALL that apply

**A**	Poor general performance of NAVA	○
**B**	Lack of experience / insufficient skill set / lack of confidence	○
**C**	Insufficient evidence base	○
**D**	Inadequate clinical guidelines	○
**E**	Increased workload	○
**F**	The need to replace the existing NG catheter	○
**G**	Cost	○
**H**	Resistance from senior clinicians / management	○
**I**	No barriers	
**J**	Other (Please state)	○

27. What do you consider to be the **MAIN** barrier from the list above?

    Please enter ONE letter from the list above. **Record NA if you answered 'I'**

28. What initiative do you think would most help the acceptance and use of NAVA at KCH? ***Tick ONE option***

Well conducted research	Improved training	Improved clinical guidelines and protocol	The creation of a 'Super User' group	NAVA catheters in all patients	Other (please write below)
o	o	o	o	o	o

Other

29. Assuming no contraindications to either mode, which mode would you prefer when initiating ventilator weaning? ***Tick one option***

NAVA	Pressure Support	No preference	Other
o	o	o	o

Other

30. Please indicate your general feelings towards NAVA on the scale below. ***Tick ONE option***

Strongly dislike NAVA	Moderately dislike NAVA	Slightly dislike NAVA	Ambivalent	Slightly like NAVA	Moderately like NAVA	Strongly like NAVA
o	o	o	o	o	o	o

31. Thinking about your experience, how frequently is the NAVA mode 'switched' to the PS mode?

Never	Rarely	Sometimes	Often	Always	Don't know / NA
o	o	o	o	o	o

32. In your experience, what are the main reasons for switching from NAVA to the PS mode? ***Tick all that apply***

**A**	Lack of knowledge / unfamiliarity with the NAVA technology	o
**B**	Personal clinician preference	o
**C**	Problems with the diaphragm signal	o
**D**	Inappropriate pressures or volumes	o
**E**	High respiratory rate	o
**F**	Inadequate gas exchange	o
**G**	Loss of NG catheter	o
**H**	Don't know	o

**I**	Other (Please state)	○

33. What is the main reason that the NAVA mode may be switched to the PS mode?

    Please enter ONE letter from the list above. Record NA if you answered 'J'

34. What do you consider to have had the biggest impact on your personal practice and views towards NAVA?

    Tick ONE option

**Published research**	**Your own experience**	**Colleague / peer recommendation**	**Other (please state below)**
○	○	○	○
Other			

35. Please indicate your level of agreement with the following statements:

	**Strongly disagree**	**Disagree**	**No opinion**	**Agree**	**Strongly agree**	**NA**
A: NAVA is safe	○	○	○	○	○	○
B: The NAVA mode is clinically effective	○	○	○	○	○	○
C: Diaphragm monitoring is clinically effective	○	○	○	○	○	○
D: Ventilator dsynchrony is a clinically significant issue	○	○	○	○	○	○
E: The NAVA mode is more difficult to use than PS	○	○	○	○	○	○

RESTUS / NAVA RESEARCH

The current RESTUS feasibility study aims to investigate our use of NAVA in prolonged weaning to help us to understand if a larger trial is possible.

36. Are you broadly supportive of the aim of the current research study as stated above?

**Yes**	**No**	**Don't know**
○	○	○

37. Have you cared for a patient who was recruited to the NAVA/ RESTUS trial?

Yes	No	Can't remember / don't know
○	○ (Go to Question 39)	○

38. If you have cared for a patient in the RESTUS / NAVA study, how acceptable did you find the research protocol and research process?

Not acceptable	Slightly acceptable	Moderately acceptable	Very acceptable	Completely acceptable	Don't know
○	○	○	○	○	○

a. Please comment below.

………

FINAL COMMENTS

39. Thank you for completing the survey. Please feel free to add any comments or suggestions below.

………

………

………
