# Supplementary material for: Factors affecting the use of neurally adjusted ventilatory assist in the adult critical care unit: a clinician survey
Source: BMJ Open Respir Res. 2020 Dec 8;7(1):e000783. doi: 10.1136/bmjresp-2020-000783 (PMC7725091; doi:10.1136/bmjresp-2020-000783)
Supplement: Supplementary data [file bmjresp-2020-000783supp002.pdf]

Supplemental file 2: Response rates and additional data

Manuscript title

Factors affecting the use of Neurally Adjusted Ventilatory Assist in the adult critical care unit: A clinician survey

Corresponding Author

Daniel Hadfield. Email: daniel.hadfield@nhs.net.

Contents

Table 1. Response rates for each question 2
Figure S1. Familiarity with the RESTUS trial and aspects of weaning care. 3
Figure S2. Application of Edi monitoring 4
Figure S3. Staff level of agreement with four statements 5
Figure S4. General feelings towards NAVA 6
Figure S5. Perceived clinical performance of NAVA versus PSV 6
Figure S6. Ease of use, NAVA versus PSV 7
Figure S7. Workload associated with NAVA by profession 8
Figure S8. Confidence of NAVA use 9
Figure S9. Perceived advantages of NAVA 10
Figure S10. Perceived disadvantages of NAVA 11
Figure S11. Barriers to the acceptance and implementation of NAVA 12
Figure S12. Frequency of mode cross-over 13
Figure S13. Reasons for mode cross-over 14
Figure S14. Initiatives to help with clinician acceptance of NAVA 15

Table 1. Response rates for each question

Item number	Number of responders	Item number	Number of responders
**1**	301	**19c**	120
**2**	247	**19d**	123
**3**	50	**19e**	131
**4**	285	**19f**	131
**5**	300	**19g**	126
**6**	301	**19h**	110
**7**	295	**20**	190
**8**	231	**21**	190
**9**	294	**22**	182
**10a**	293	**23**	189
**10b**	294	**24**	173
**10c**	293	**25**	188
**10d**	292	**26**	183
**10e**	291	**27**	177
**11**	294	**28**	167
**12**	287	**29**	181
**13**	29	**30**	179
**14**	193	**31**	182
**15a**	191	**32**	180
**15b**	193	**33**	168
**15c**	193	**34**	174
**15d**	193	**35a**	181
**15e**	190	**35b**	181
**15f**	192	**35c**	181
**15g**	192	**35d**	179
**15h**	192	**35e**	181
**16**	191	**36**	180
**17**	192	**37**	121
**18**	193	**38**	125
**19a**	116	**39**	42
**19b**	121		

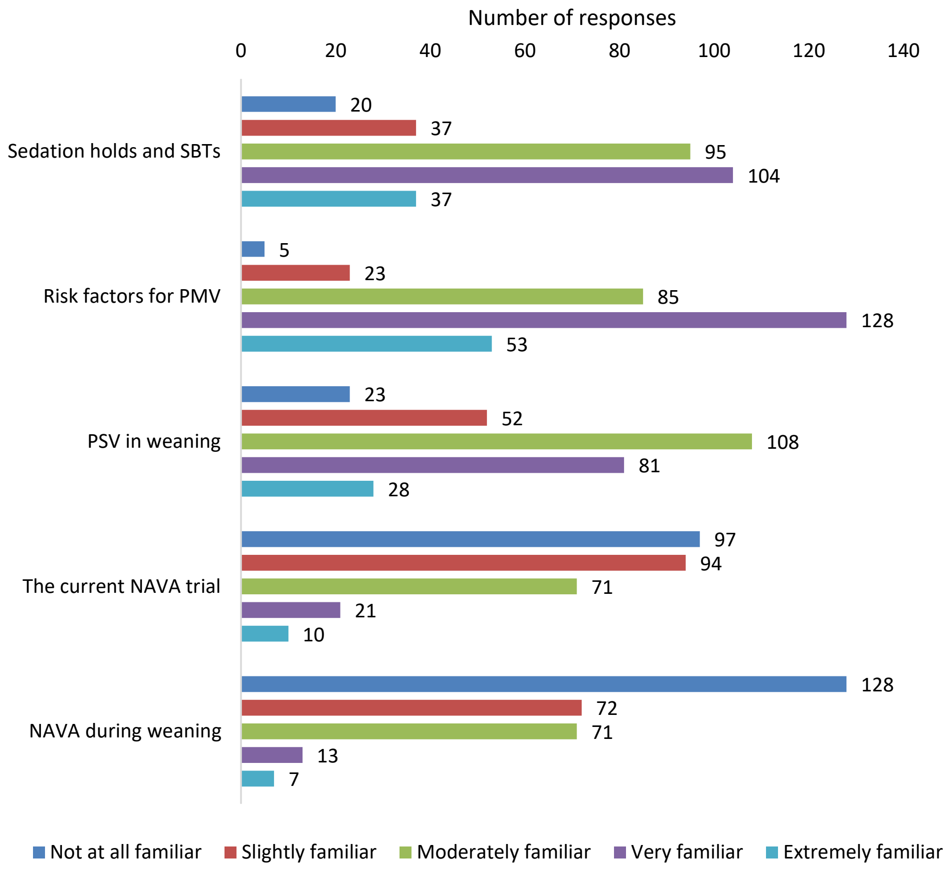

Figure S1. Familiarity with the RESTUS trial and aspects of weaning care.

Please indicate your familiarity with the following: A. Evidence supporting the use of sedation holds and spontaneous breathing trials (SBTs); response rate = 293

B. Risk factors for prolonged MV (PMV); response rate = 294

C. Evidence supporting the use of PSV during weaning; response rate = 292

D. The current NAVA trial; response rate = 292

E. Evidence supporting the use of NAVA during weaning; response rate = 291

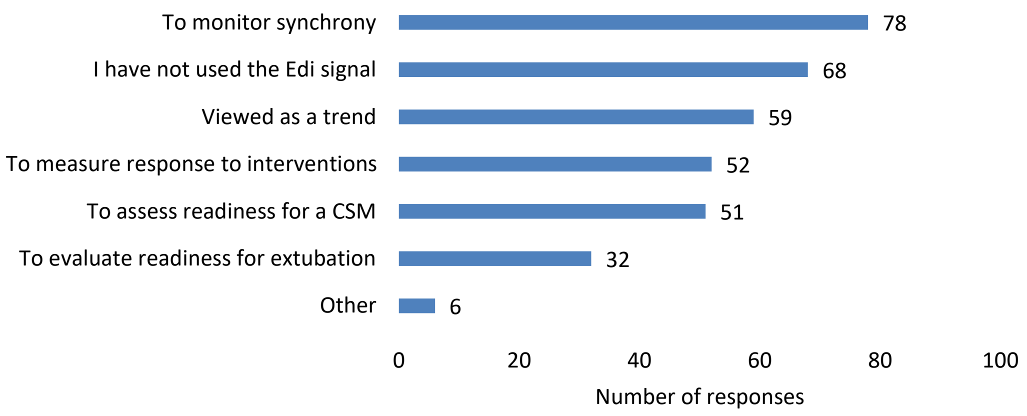

Figure S2. Application of Edi monitoring

How have you used the Edi (electrical activity of the diaphragm) signal? Participants could select multiple items. Response rate: 191. 'Other' responses (n=6), were categorised as 'Invalid' (n=5) and one referred to the use of Edi monitoring in post-extubation support. CSM = Continuous Spontaneous Mode (NAVA or PSV)

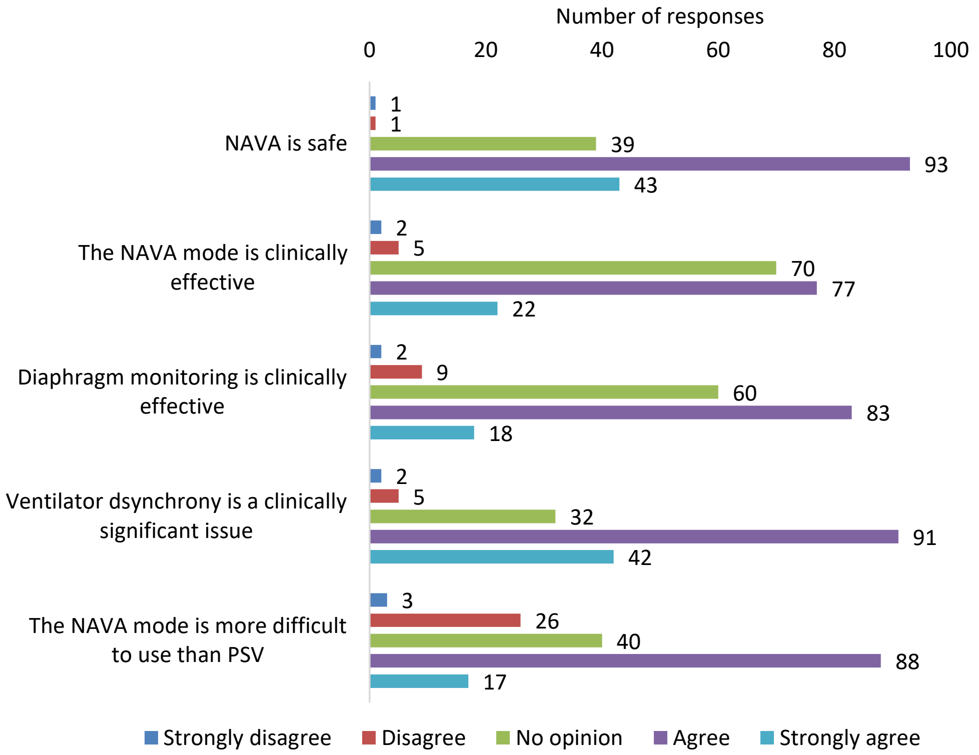

Figure S3. Staff level of agreement with four statements

Please indicate your level of agreement with the following statements: A. NAVA is safe; total responses 181; NA = 4

B. The NAVA mode is clinically effective; total responses 181; NA = 5

C. Diaphragm monitoring is clinically effective; total responses 181; NA = 9

D. Ventilator dysynchrony is a clinically significant issue; total responses 179; NA = 7

E. The NAVA mode is more difficult to use than PSV; total responses 181; NA = 7

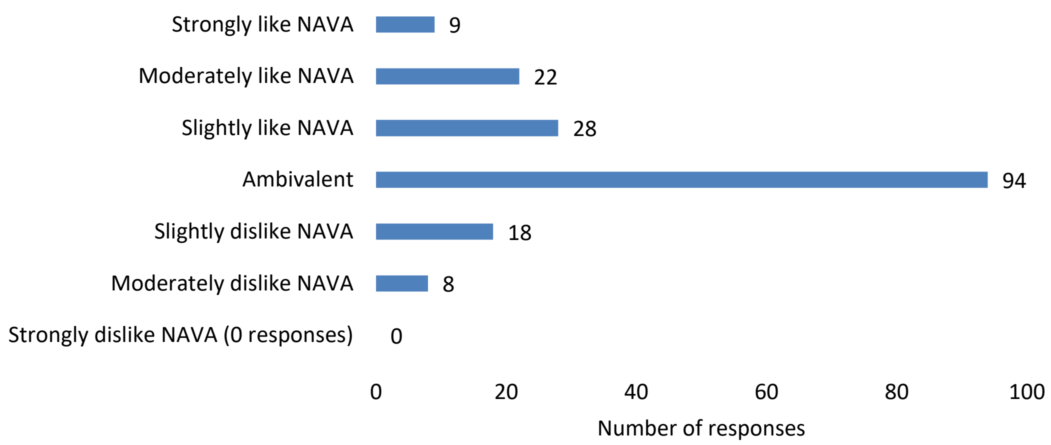

Figure S1. General feelings towards NAVA

Please indicate your general feelings towards NAVA.Total respondents 179. Participants could select one option only. Total respondents = 179

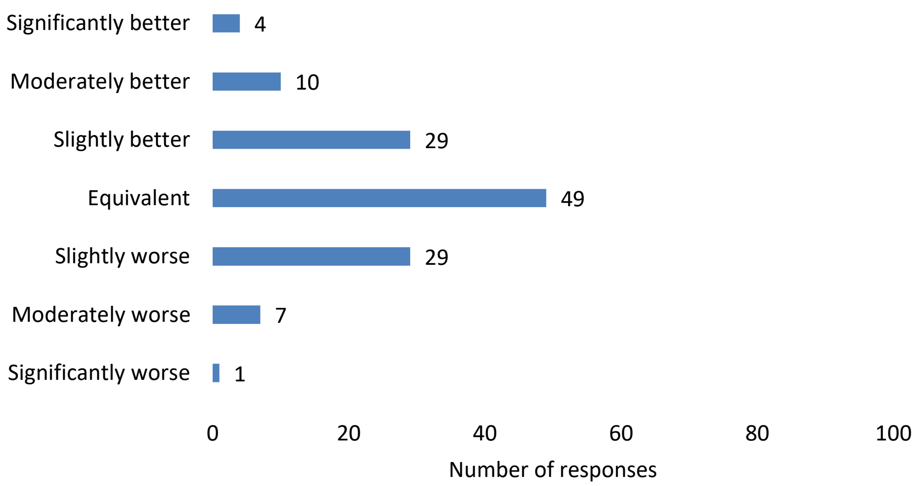

Figure S5. Perceived clinical performance of NAVA versus PSV

In your experience, how did NAVA perform clinically in comparison to Pressure Support? Total respondents = 190. Participants could answer one option only. ‘Don’t know’ (n=61)

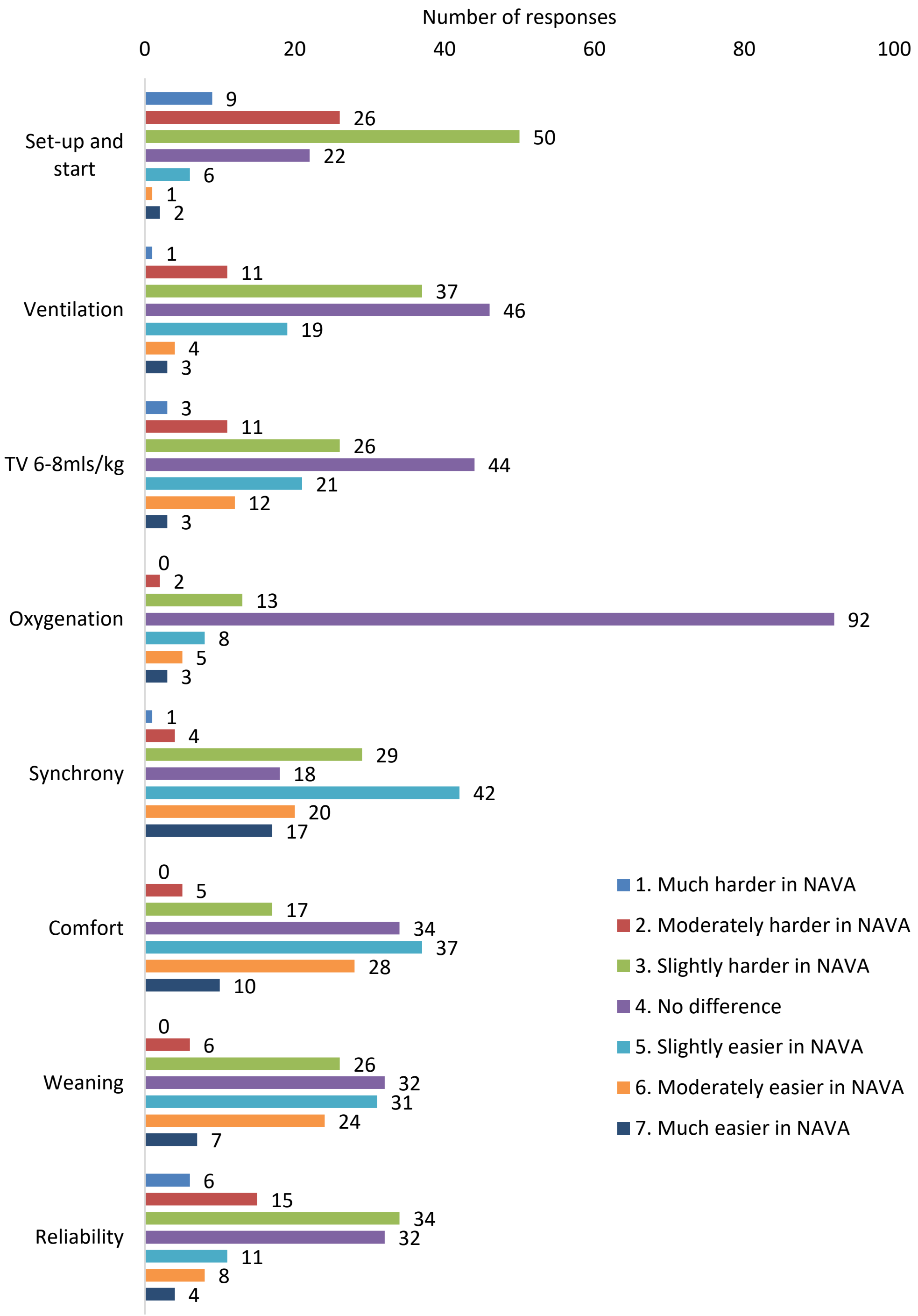

Figure S6. Ease of use, NAVA versus PSV

How easy is it to achieve the following aspects of ventilation practice when using the NAVA mode compared to the PSV mode? Response rates and 'Not Applicable' (NA) responses:

A. Set-up and start; response rate 116, NA 75

B. Ventilation (adequate minute volume and CO_2 clearance); response rate 121, NA 70

C. Lung protection (achieving tidal volumes of 6-8mls/kg); response rate 120, NA 71

D. Oxygenation; response rate 123, NA 68

E. Synchrony; response rate 131, NA 60

F. Patient comfort; response rate 131, NA 61

G. Weaning; response rate 126, NA 66

H. Maintaining the mode without 'switching' (reliability); response rate 110, NA 82

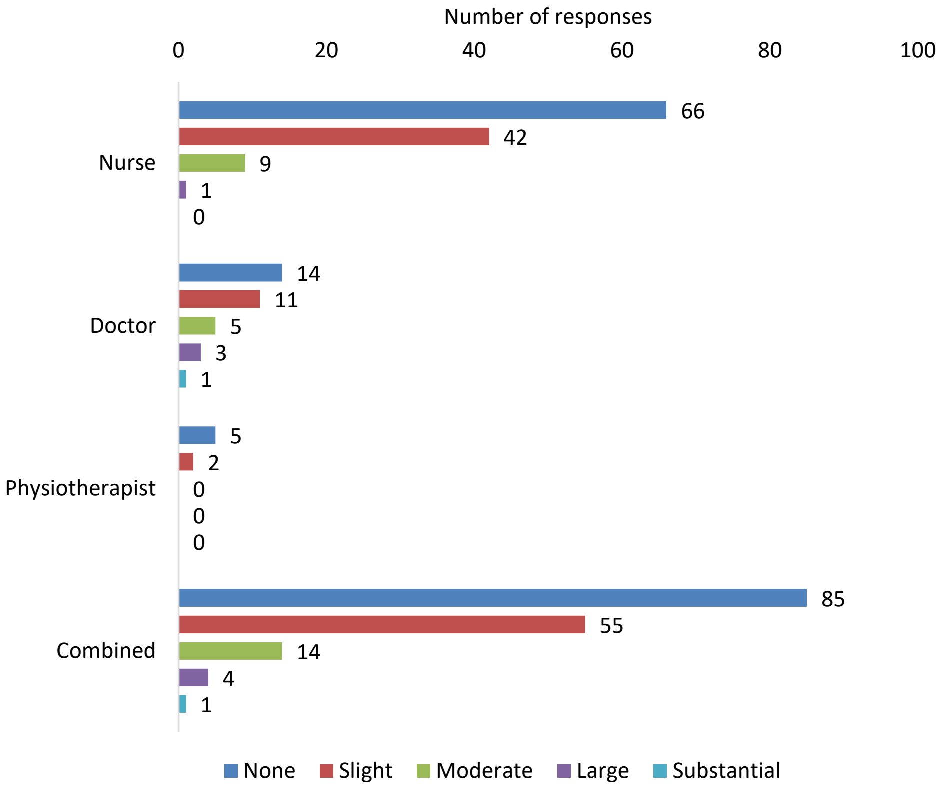

Figure S7. Workload associated with NAVA by profession

In your experience, is there an increase in your personal workload associated with the use of NAVA? Total respondents 188, comprised of 118 nurses, 34 doctors and 7 physiotherapists. Don't know/NA responses (n=29)

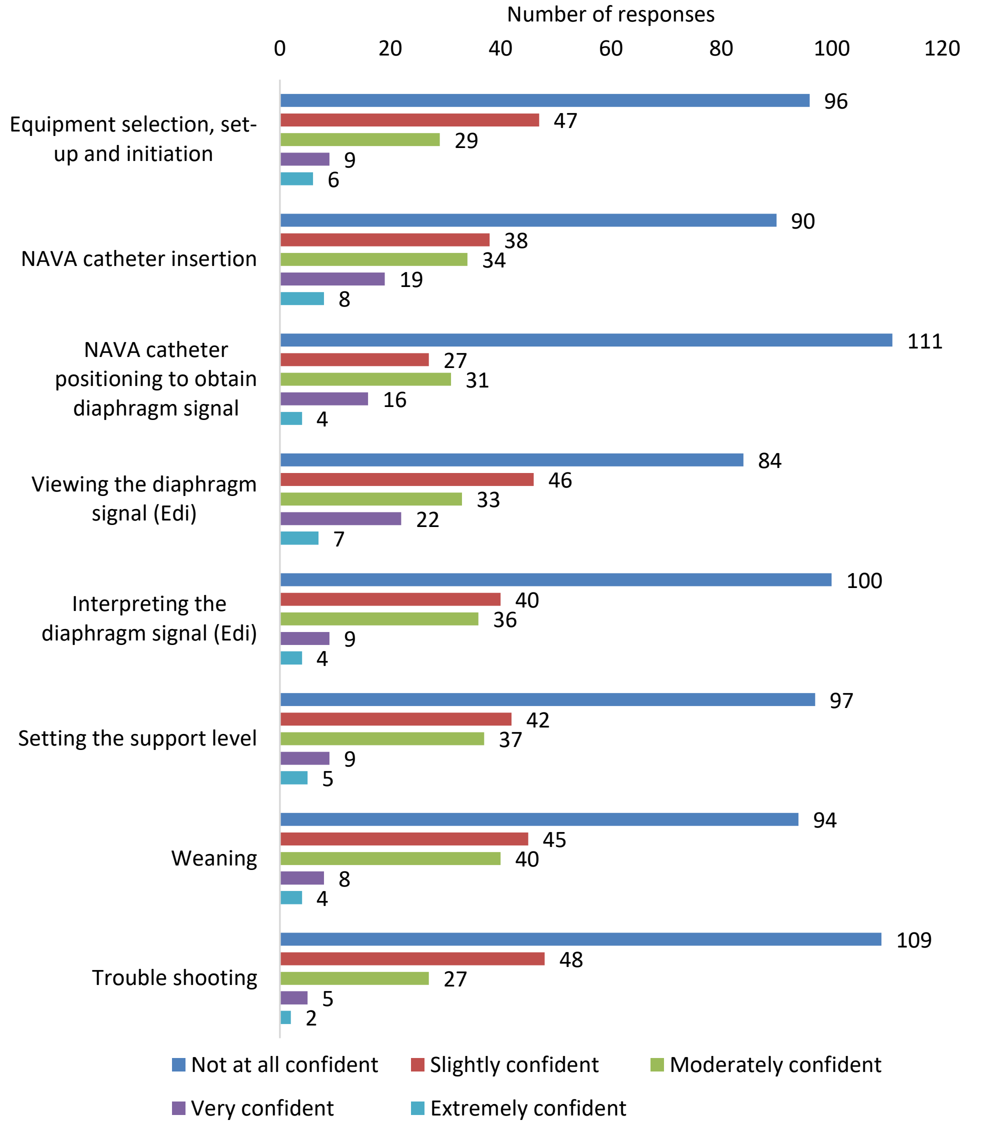

Figure S2. Confidence of NAVA use

How confident are you in performing the following NAVA related tasks? Response rates and 'Not Applicable' (NA) responses:

A: Equipment selection, set-up and initiation; response rate 191, NA 4

B: NAVA catheter insertion; response rate 193, NA 4

C: NAVA catheter positioning to obtain diaphragm signal; response rate 193, NA 4

D: Viewing the diaphragm signal (Edi); response rate 193, NA 1

E: Interpreting the diaphragm signal (Edi); response rate 190, NA 1

F: Setting the support level; response rate 192, NA 2

G: Weaning; response rate 192, NA 1

H: Trouble shooting; response rate 192, NA 1

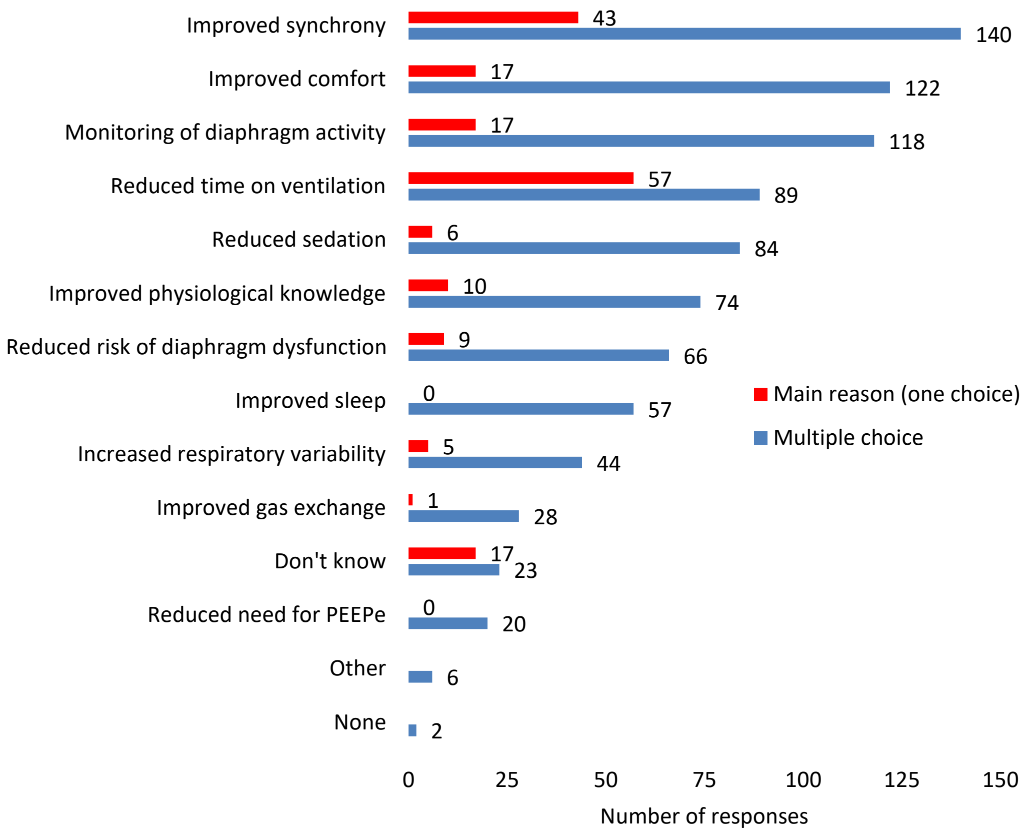

Figure S9. Perceived advantages of NAVA

What do you consider are the potential clinical benefits of using NAVA technology in comparison to PSV? Participants could select multiple options (blue line). Other responses were categorised as ‘invalid’ (n=5) and ‘Monitoring of diaphragm activity’ (n=1). Response rate: 190. Participants were also asked ‘What do you consider to be the one most important potential clinical benefit from the list above?’ Participants were asked to tick one option only. Response rate = 182. Two ‘Other’ responses were categorised as ‘invalid’.

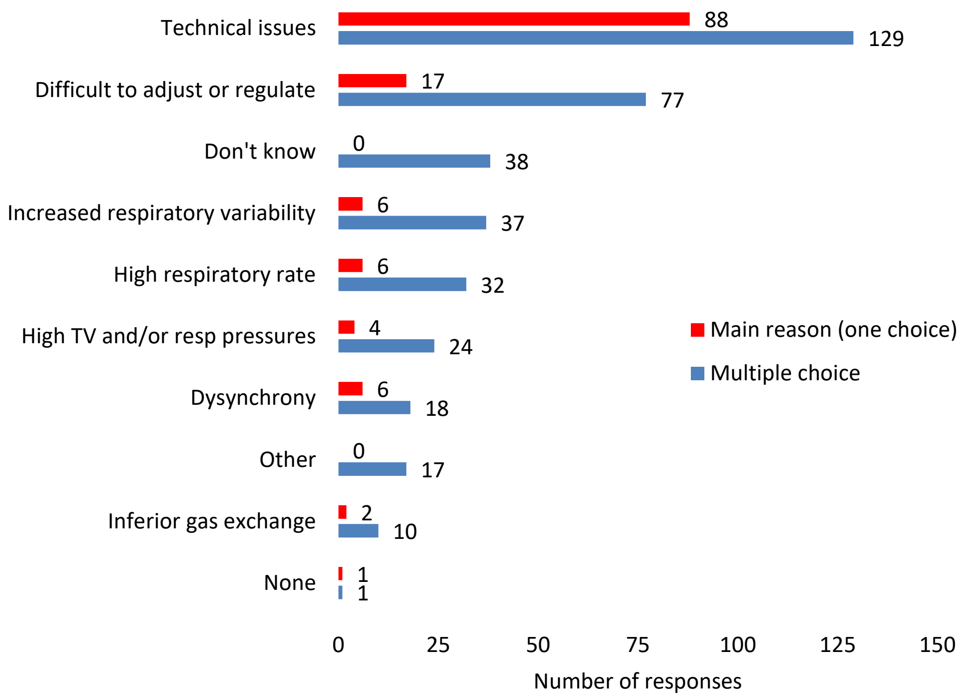

Figure S10. Perceived disadvantages of NAVA

What do you consider are the potential clinical disadvantages of using NAVA in comparison to Pressure Support? Participants could select multiple items. Participants were informed that the response 'Technical issues' included signal quality, equipment malfunction, reliability. Response rate: 189. Free text other responses referred to difficulty with NAVA catheter connections (n=1), increased complexity in trouble shooting (n=1), and lack of staff training/familiarity (n=2). Participants were also asked 'What do you consider is the one most important clinical disadvantage?'. Response rate: 173. Participants were asked to tick one option only (red line). Other responses included 'NA' (n=8), lack of knowledge/familiarity (n=6) and the possibility of accidental NAVA catheter removal (n=1)

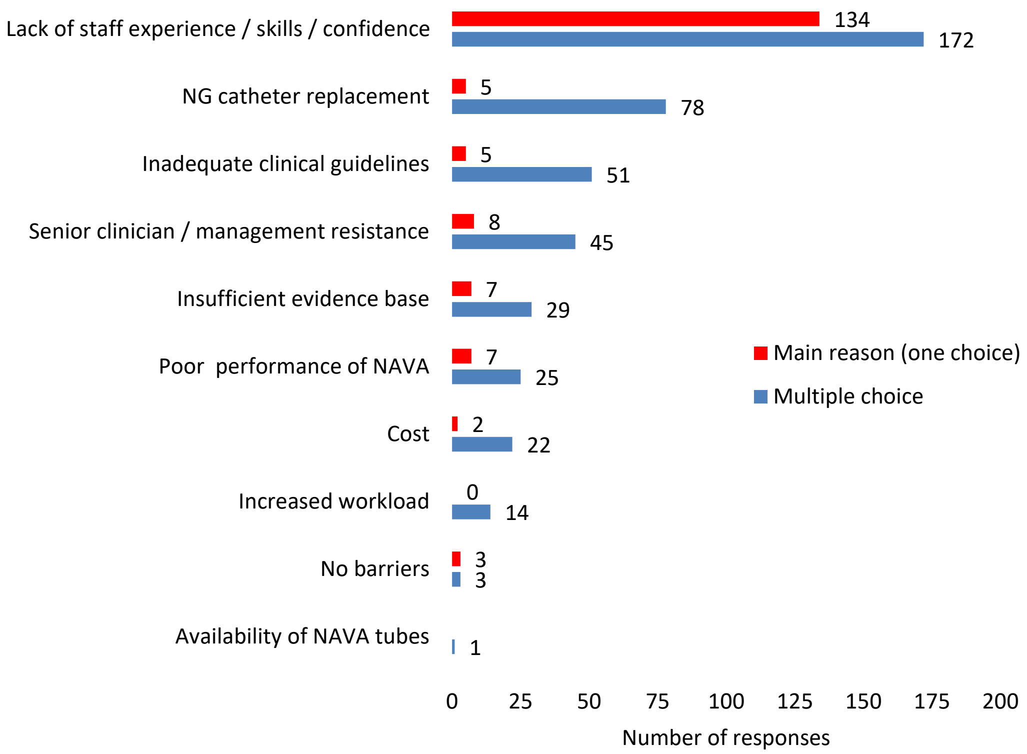

Figure S11. Barriers to the acceptance and implementation of NAVA

What do you consider are the main barriers to the acceptance and implementation of NAVA?Total respondents = 183. Participants could select multiple options (blue line). Other responses (n=12) were categorised post hoc as ‘Lack of staff experience / skills confidence’ (n=4), availability of NAVA tubes (n=1), ‘Poor performance of NAVA’ (n=1), and ‘invalid’ (n=6). Participants were also asked, **‘**What do you consider to be the main barrier from the list above?’. Participants were asked to select one option only (red line). Total respondents = 177. ‘Other’ responses (n=8) were categorised post hoc to ‘Lack of staff experience / skills confidence’ (n=2) and ‘invalid’ (n=6).

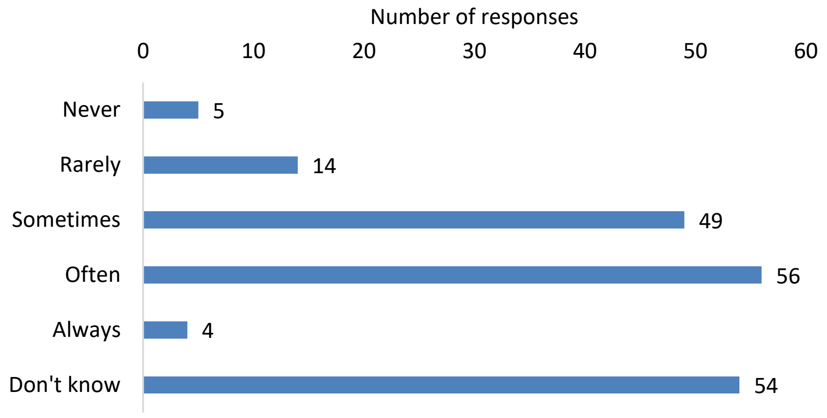

Figure S12. Frequency of mode cross-over

Thinking about your experience, how often is the NAVA mode 'switched' to the PSV mode? Total respondents = 182. Participants could select one option only

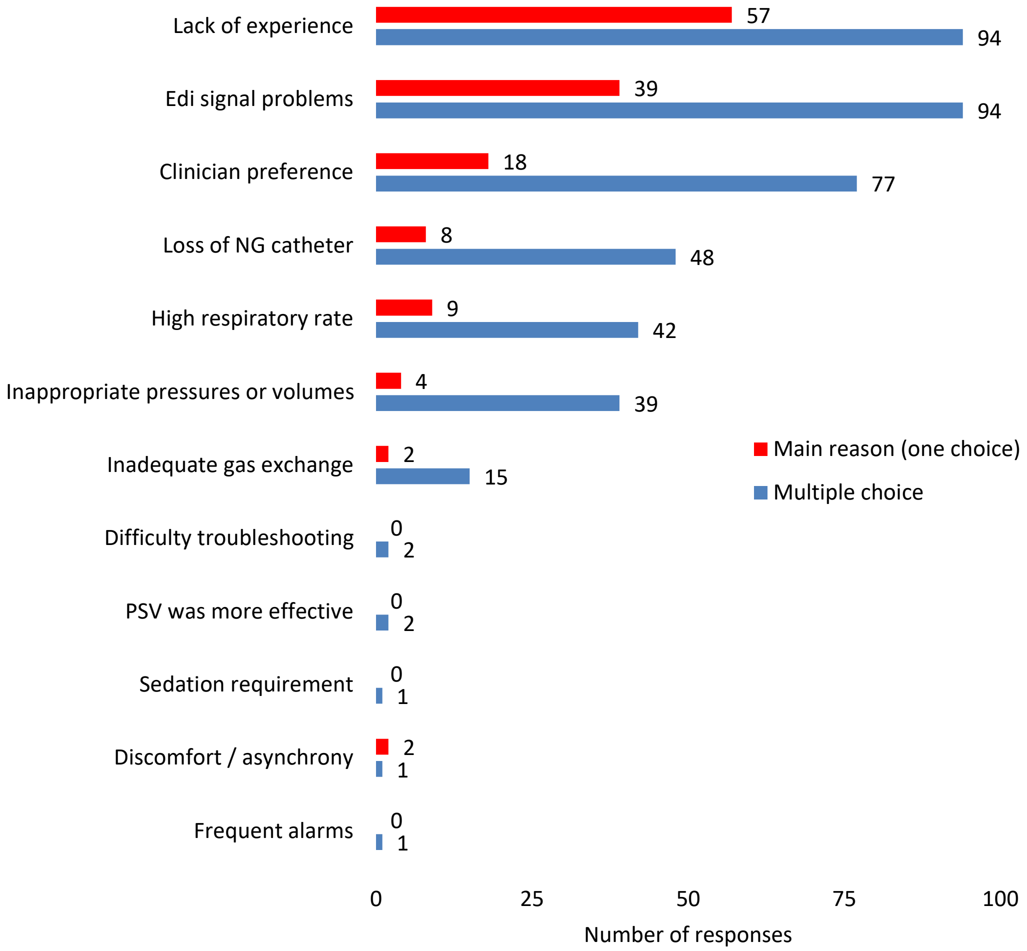

Figure S3. Reasons for mode cross-over

In your experience, what are the main reasons for switching from NAVA to the PSV mode? Total respondents = 180. Participants could select multiple options (blue line). Participants were also asked to select what they considered to be the main reason (one option only) for switching from NAVA to the PSV mode (red line); total respondents = 168. 'Other' responses (n=6) were categorised as 'Sedation requirement' (n=1), 'Lack of experience' (n=3), 'Patient discomfort / dysynchrony' (n=2), PSV more effective (n=3), difficulty troubleshooting (n=2), 'invalid' (n=4).

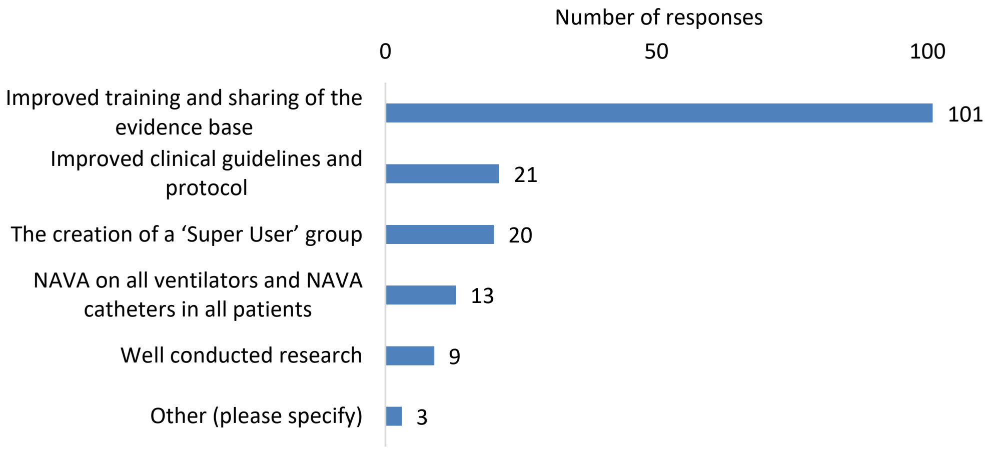

Figure S14. Initiatives to help with clinician acceptance of NAVA

What initiative do you think would most help the acceptance and use of NAVA at KCH? Participants could answer one option only. ‘Other’ responses were classified as invalid
